# Endogenously generated 2-aminoacrylate inhibits motility in *Salmonella enterica*

**DOI:** 10.1038/s41598-017-13030-x

**Published:** 2017-10-11

**Authors:** Andrew J. Borchert, Diana M. Downs

**Affiliations:** Department of Microbiology, University of Georgia, Athens, GA USA

## Abstract

Members of the broadly distributed Rid/YER057c/UK114 protein family have imine/enamine deaminase activity, notably on 2-aminoacrylate (2AA). Strains of *Salmonella enterica*, and other organisms lacking RidA, have diverse growth phenotypes, attributed to the accumulation of 2AA. In *S*. *enterica*, 2AA inactivates a number of pyridoxal 5’-phosephate(PLP)-dependent enzymes, some of which have been linked to the growth phenotypes of a *ridA* mutant. This study used transcriptional differences between *S*. *enterica* wild-type and *ridA* strains to explore the breadth of the cellular consequences that resulted from accumulation of 2AA. Accumulation of endogenously generated 2AA in a *ridA* mutant resulted in lower expression of genes encoding many flagellar assembly components, which led to a motility defect. qRT-PCR results were consistent with the motility phenotype of a *ridA* mutant resulting from a defect in FlhD_4_C_2_ activity. In total, the results of comparative transcriptomics correctly predicted a 2AA-dependent motility defect and identified additional areas of metabolism impacted by the metabolic stress of 2AA in *Salmonella enterica*. Further, the data emphasized the value of integrating global approaches with biochemical genetic approaches to understand the complex system of microbial metabolism.

## Introduction

Microbes inhabit environments that are often dynamic with respect to nutrient availability and the presence of chemical and/or physical stresses. A hallmark of microbes is their ability to respond to changing conditions by altering their metabolic network and/or physical characteristics to survive and thrive under new conditions^1,2^. A common response to stresses and/or changing environmental conditions is the coordinated regulation of genes that ameliorate the stress and exploit changing nutrient circumstances^3–5^. Numerous examples of global responses to environmental stress and nutrient availability that are mediated by transcriptional regulation have been described^1,2^. Characterization of these responses has contributed to our understanding of not only metabolic strategies but mechanisms of regulation, effector binding, and gene function. Metabolism can be regulated at many levels, but perturbations in the metabolic network are ultimately expected to, directly or indirectly, impact the transcriptome. Thus, the global expression pattern of a microbe can be used to decipher the environment perceived by the cell and understand conditions both internal and external to the organism^6–8^.

Metabolic imbalance caused by inhibition of a specific enzyme, for instance by exposure to antimicrobial agents or toxic metabolites, often result in transcriptional changes that are triggered as a consequence of the resultant metabolic imbalance^9–12^. In this scenario, if multiple insults generated similar or overlapping transcriptional profiles, it would indicate the perturbations ultimately affected the same part of metabolism. For instance, in a *Bacillus subtilis* strain harboring a *purA* mutation that diminishes flux toward adenine biosynthesis, the cell senses a purine limitation and attempts to overcome it via de-repression of the PurR regulon^13^. Treatment of *Bacillus subtilis* with the dihydrofolate reductase (FolA; EC 1.5.1.3) inhibitor, Trimethoprim, causes a disruption of C_1_ unit transfer in the cell, resulting in a bottleneck in purine biosynthesis at the AICAR transformylase (PurH; EC 2.1.2.3) enzyme. In this case, the cell perceives a purine limitation and attempts to overcome it via de-repression of the PurR regulon^14^. Thus, the shared features of the transcriptional response to both perturbations indicated that the perceived stress in each case was purine limitation.

**Figure 1:**
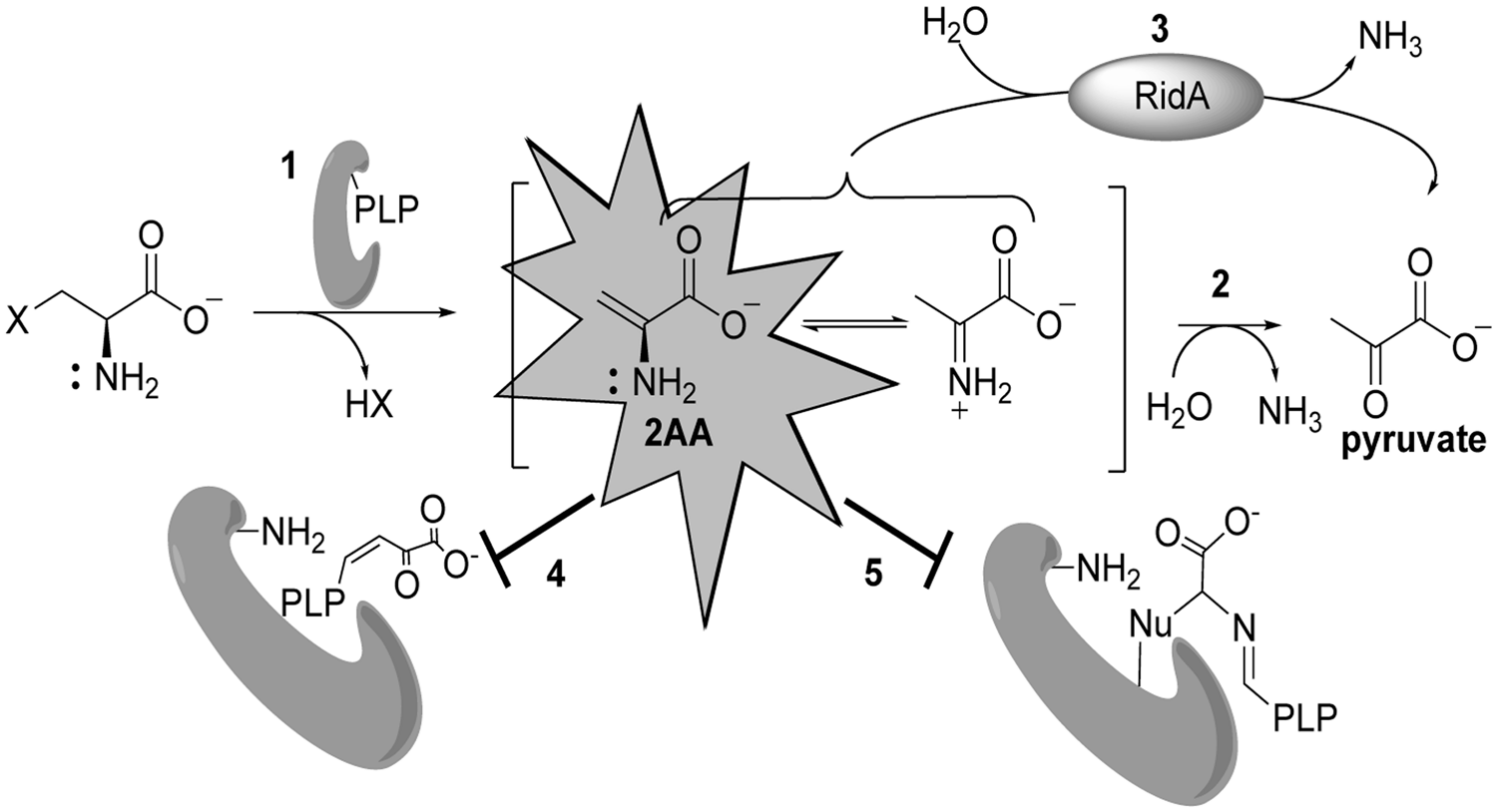
RidA paradigm of 2AA stress in *S*. enterica. Multiple pyridoxal 5′-phosphate (PLP)-dependent enzymes can catalyze β-elimination reactions of 3-carbon alpha amino acids containing favorable leaving groups on the beta-carbon (1). This generates the reactive enamine intermediate 2-aminoacrylate (2AA), which goes into solution. 2AA has three possible fates following its release from the active site: First, 2AA can tautomerize to its imine form, producing 2-iminiopropanoate, which is non-enzymatically hydrolyzed, forming the stable keto-acid pyruvate (2). Second, RidA catalyzes the deamination of 2AA to pyruvate (3). Finally, 2AA can attack the internal aldimine of various PLP enzymes, irreversibly damaging the target enzyme. 2AA attack results in the formation of an inactive pyruvate-PLP adduct (4) or covalent modification of the enzyme by the 2AA-PLP aldimine (5).

In *Salmonella enterica*, accumulation of the reactive enamine species 2-aminoacrylate (2AA) perturbs the metabolic network by partially inactivating a number of PLP-dependent enzymes, which results in growth phenotypes^15–19^. 2AA is generated as a mechanistic intermediate of multiple PLP-dependent enzymes in central metabolic pathways (e.g., serine/threonine dehydratases EC 4.3.1.19)^20,21^. Thus far, the validated targets of 2AA *in vivo* are PLP enzymes where the enamine attacks the internal aldimine and results in the covalent modification of the PLP cofactor. Depending on the mechanism of attack, an inactive pyruvate-PLP adduct is formed in the active site, or the enzyme itself forms an irreversible bond with the modified PLP^17,19,22^. In either scenario, the affected enzyme is irreversibly damaged, and flux through the pathway to which it belongs is constrained. A summary of the described RidA paradigm of 2AA stress in *S*. *enterica* is provided in Fig. 1. Most bacteria, including *S*. *enterica* and *E*. *coli*, have multiple RidA paralogs, but to date no physiological role has been identified for these proteins.

Members of the RidA subfamily of the Rid/YER057c/UK114 protein superfamily, are enamine deaminases that quench 2AA and thus prevent the accumulation of this metabolite *in vivo* ^16,23^. The combination of *in vitro* and *in vivo* studies in *S*. *enterica* showed that at least the PLP-dependent enzymes serine hydroxymethyltransferase (GlyA; EC 2.1.2.1), branched-chain amino acid aminotransferase (IlvE; EC 2.6.1.42) and alanine racemase (Alr/DadX; EC 5.1.1.1) were targets of free 2AA *in vivo* and their activity is between 40–80% decreased in strains lacking RidA^17,19,22,24,25^. The presence of RidA proteins across all domains of life supports a conserved role for this family in quenching reactive metabolites, including 2AA, to prevent damage to metabolic components.

The current understanding of 2AA targets *in vivo* is the result of biochemical genetic studies, primarily in *S*. *enterica*. Although powerful, this approach demanded that the damage caused by 2AA generated a detectable growth phenotype. It was therefore likely that the impact of free 2AA on the metabolic network was broader than what has been defined by this approach. Additionally, the impact that 2AA damage of known targets has on the global network configuration remains unexplored. For example, while it is known that the growth defect of an *S*. *enterica ridA* mutant grown in minimal medium is caused by 2AA damage to serine hydroxymethyl transferase (GlyA; EC 2.1.2.1), it is unclear how the cell perceives the resulting reduction in C_1_ unit production^24^. The current study was initiated to generate a global view of metabolic changes that result from the accumulation of 2AA in *S*. *enterica*. The premise of this work was that a transcriptional profile reflects expression changes that result, directly or indirectly, from perceived changes in the internal or external environment^6,8,26^.

## Results

### Deletion of *ridA* alters the global transcription profile

The transcriptomes of a *S*. *enterica ridA* mutant and the isogenic wild-type strain were defined after growth on minimal glucose medium to provide a snapshot of the global metabolic state of the cells. The transcriptome data were viewed with two questions in mind; i) are previously characterized metabolic defects caused by elevated 2AA discoverable, and ii) do the data generate new insights about the global impact of 2AA accumulation?

**Figure 2:**
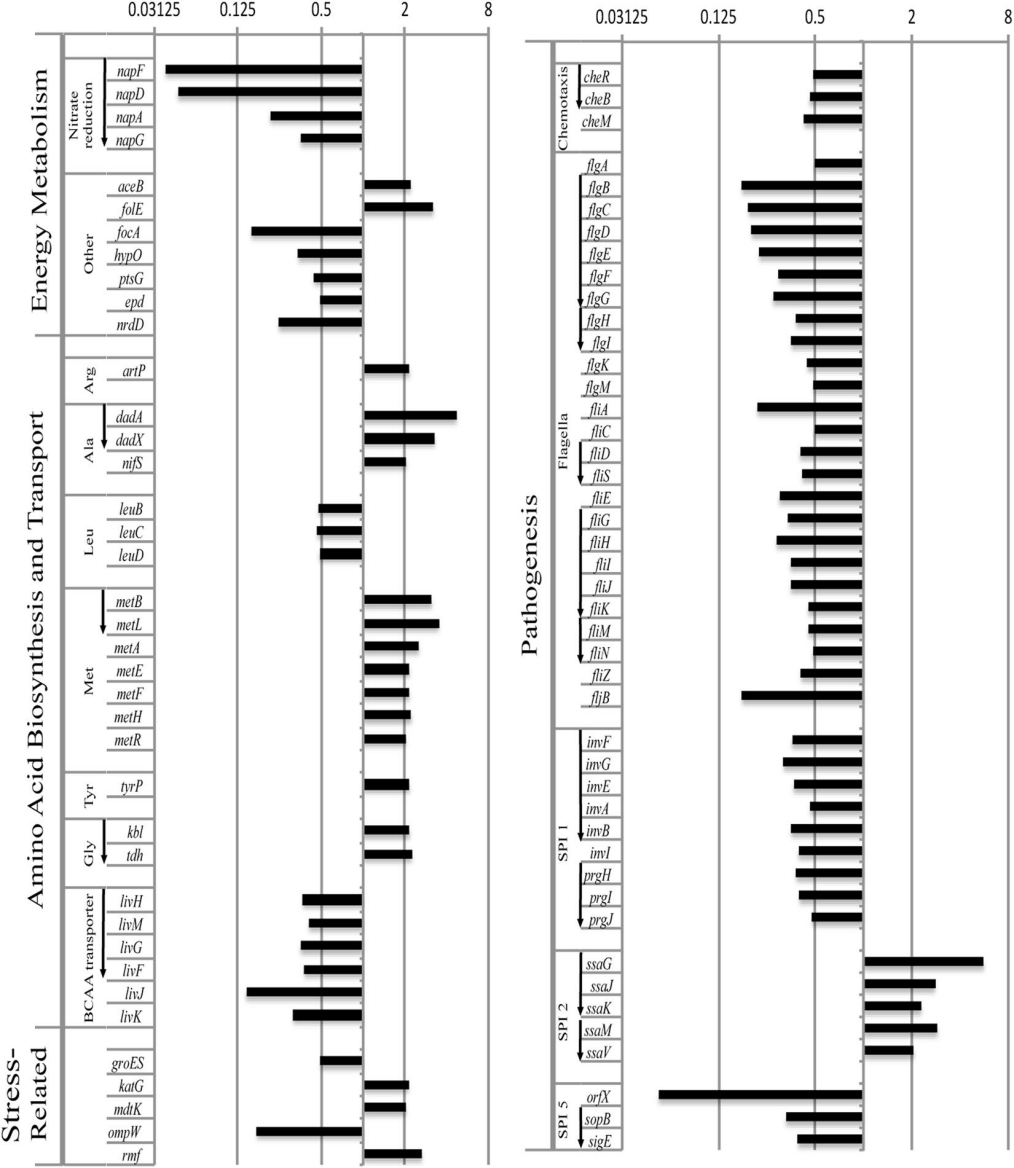
Gene expression differs between a *ridA* mutant and wild type strain. Summary of relative expression levels of genes in *S*. Typhimurium LT2 found to differ significantly (FDR < 0.05, Fold-change >2) between *ridA* and wild-type strains. A value of 1 indicates no detectable difference in expression, values > 1 indicate higher expression in the *ridA* mutant, and values < 1 indicate higher expression in wild-type. Arrows to the left of the gene name indicate whether that gene is contained within an operon, with the order of the operon arranged following the arrow base to the arrowhead.

Pair-wise comparison of gene expression identified 413 genes with changes (186 higher expression, and 227 lower expression) in transcript abundance between *ridA* mutant and wild-type strains, using a false discovery rate (FDR) less than 0.05 (Table S1)^27^. Of these loci, genes with no known function (STMxxxx and *yxxX* locus tags) and those that showed less than a two-fold difference in expression were excluded to generate the dataset of 84 genes shown in Fig. 2. There was no evidence that the biosynthetic genes for isoleucine (*ilv*) were de-repressed in a *ridA* mutant, despite the fact that the specific activity of isoleucine transaminase (IlvE, the last step in isoleucine synthesis) is more than 50% reduced in the absence of RidA^17,18^. These data corroborated the conclusion reached with a single reporter, that lack of RidA did not result in a starvation for isoleucine^18^. Similarly, no differential expression was simply attributable to the decreased activity of serine hydroxymethyl transferase (GlyA) found in *ridA* mutants. The transcription of *dadAX* was increased in the *ridA* mutant and potentially reflected a consequence of the decreased alanine racemase activity of DadX, caused by 2AA accumulation^22^, since increased accumulation of L-alanine may contribute to the complex transcriptional regulation of *dadAX* ^28^.

**Figure 3:**
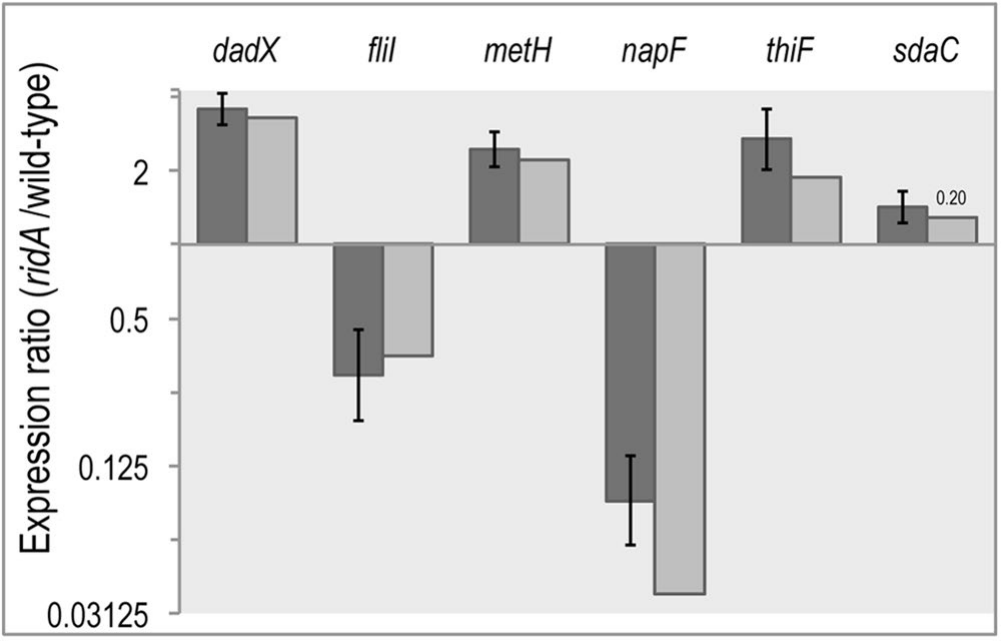
qTR-PCR supports RNAseq differential expression results for a subset of genes. Differential expression of a subset of genes for *S*. *enterica* LT2 *ridA* strain (DM3480) relative to its isogenic wild-type (DM9404) as determined by qRT-PCR (dark grey bars). The RNAseq differential expression values (light grey bars) were included as a reference for comparison. Error bars on qRT-PCR data represent the 95% confidence interval. All genes presented, excluding *sdaC*, displayed differential expression (*ridA*/wild-type) with FDR < 0.05, following RNAseq analysis; the corresponding FDR value for *sdaC* is provided above the bar representative of the RNAseq data.

Results from the global RNA-seq experiment were independently validated with real-time qRT-PCR. The relative expression level of six select genes was measured using the comparative threshold cycle (ΔΔ*CT*) method^29^ (Fig. 3). Four of the six genes chosen met the two-fold expression threshold in the RNA-seq experiment (*napF*, *metH*, *dadX*, and *fliI*), one (*thiF*) had a statistically significant difference in expression that was less than two-fold (1.88-fold, Supplementary Table 1), and the last (*sdaC*) showed no statistically significant change in gene expression between mutant and wild-type. For the six genes, a strong positive correlation between results from the qRT-PCR and global RNA-seq approaches was supported by a Pearson’s correlation coefficient of r^2^ = 0.96. Of the four genes with a greater than two-fold expression difference, only *napF* had a statistically significant (p-value < 0.05) discrepancy between the RNA-seq and qRT-PCR data. However, both approaches detected a greater than ten-fold decrease in the *napF* transcript levels in a *ridA* mutant compared to wild-type. Finally, although RNA-seq transcriptome analysis detected increased *sdaC* expression in a *ridA* strain, the expression difference did not meet the statistical cut off (FDR < 0.05). The qRT-PCR data similarly found this transcript increased in a *ridA* mutant, but the difference was statistically significant using this technique, suggesting that the DEseq algorithm, used to call differentially expressed genes from the RNAseq dataset, performed conservatively^30^.

Features of the transcriptome data beyond metabolic gene expression were noted. For instance, multiple genes in the Salmonella pathogenicity islands (SPIs) were differentially expressed in the two strains. Genes in SPI1 and SPI5 generally had lower expression and those in SPI2 had higher expression in the *ridA* mutant compared to the wild-type strain, which was reminiscent of the expression pattern during *S*. *enterica* serovar Typhimurium replication within murine macrophages^7^. This similarity was extended with the decrease in expression of the flagellar biosynthetic operons and several genes involved in chemotaxis in the *ridA* mutant.

### Transcription profiling uncovers a role for RidA in motility

**Figure 4:**
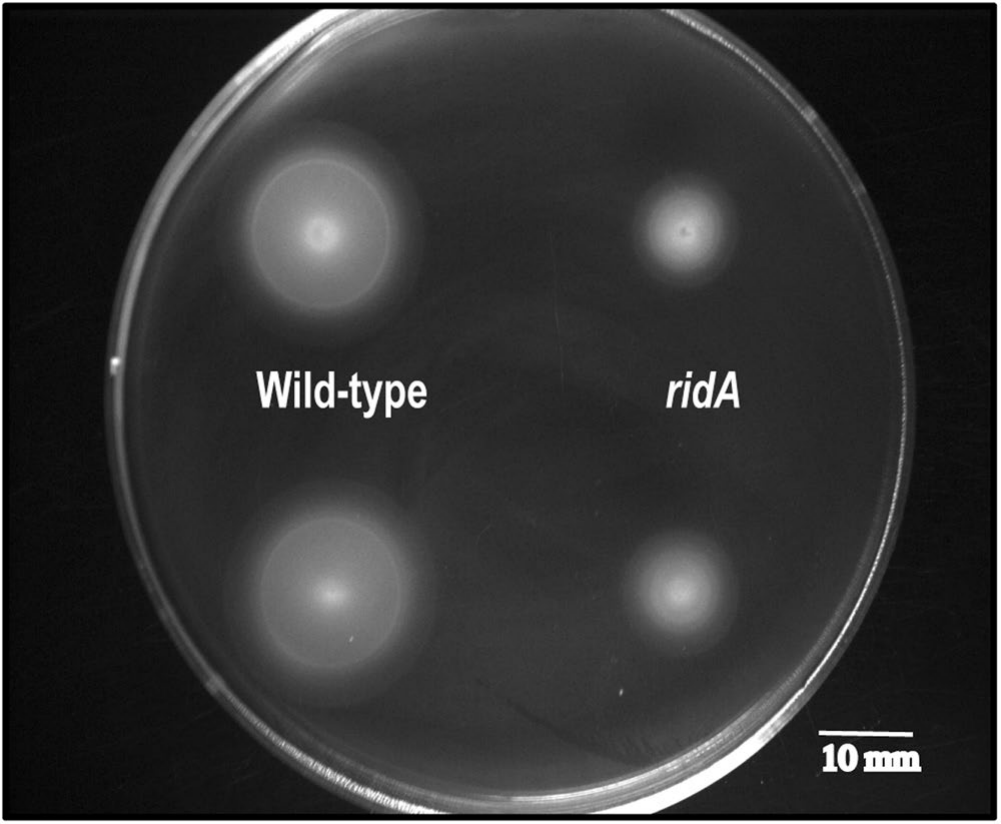
A *ridA* mutation leads to a defect in swimming motility. Representative image of swimming motility halos for *ridA* and wild-type strains grown on minimal glucose (11 mM) motility plates (0.25% agar). Following inoculation using 1 uL overnight cultures grown in LB medium, plates were allowed to incubate 20 h at 30 °C. Diameter using the outermost edge for each halo was measured to determine zone of swimming motility. Two biological replicates are shown for each strain.

Without exception, expression of the genes involved in assembly of flagella was lower, with the majority being more than two-fold lower, in a *ridA* mutant than wild-type strain. Expression of genes involved in chemotaxis, including *cheM*, *cheA* and *cheY*, was also decreased in a *ridA* mutant (2.34, 1.68, 1.78, -fold, respectively). This level of differential expression can generate a detectable motility defect^31^, and the swimming motility of the wild-type and *ridA* mutant strains was tested. As predicted by the transcript levels, the *ridA* mutant was less motile than the wild-type strain on minimal medium (Fig. 4). Quantification of the zone of swimming showed motility in *ridA* mutants was ~40% less than wildtype (Table 1), and was restored to wild-type by the expression of *ridA in trans* (Table 1, ln 5–8). It was formally possible that the slight growth defect of a *ridA* mutant on minimal medium was responsible for the *ridA* decrease in motility. The addition of glycine, which restored wild-type growth to a *ridA* strain^24^, failed to restore full motility to the mutant (Table 1, ln 3–4). This result supported the conclusion that reduced motility reflected a new consequence of a *ridA* mutation.

**Table 1:** Swimming motility is decreased in *ridA* mutant strains.

	Strain	Relevant genotype	Addition	Swim zone (mm)	% motility
1	DM9404	wild-type	None	19 ± 2	100
2	DM3480	*ridA*	None	11 ± 1	58
3	DM9404	wild-type	Gly	18 ± 2	100
4	DM3480	*ridA*	Gly	14 ± 1	78
5	DM15418	pCV1	L-ara	20 ± 1	100
6	DM15419	*ridA* pCV1	L-ara	14 ± 1	70
7	DM15420	pDM1439	L-ara	22 ± 1	100
8	DM15421	*ridA* pDM1439	L-ara	21 ± 1	95

The diameter of motility halos was determined on minimal glucose medium with 0.25% agar. When indicated, L-glycine (0.67 mM), or L-arabinose (0.2%) was present in the media. Media contained ampicillin (7.5 ug/mL) when plasmid-containing strains were used. Values shown as the average diameter and standard deviation (SD) of the growth zone of four biological replicates incubated for 20 h at 30 °C. The data for each biological replicate was the average of two technical replicates. Percent motility is the ratio of the *ridA*-/*ridA*+ isogenic strains in each case.

### Accumulation of 2-aminoacrylate (2AA) generates a motility defect

Metabolic phenotypes of a *ridA* mutant have been attributed to the accumulation of 2-aminoacrylate, which is generated by the serine/threonine dehydratase IlvA, using serine as a substrate^17^. Three lines of evidence supported the role of IlvA generated 2AA in compromising motility of a *ridA* mutant (Table 2). First, exogenous threonine restored motility (ln 1–4), presumably by outcompeting serine for the active site of IlvA, as described for other *ridA* mutant phenotypes^16,32^. Second, addition of isoleucine restored motility (ln 5,6), presumably by allosteric inhibition of IlvA^33^, that decreased the formation of 2AA^25^. The latter conclusion was further supported by strains carrying the *ilvA219* allele, which encodes an IlvA_L447F_ variant, that is resistant to allosteric inhibition by isoleucine^9^. When the *ilvA219* allele replaced wild-type *ilvA*, data in Table 2 (ln 7–10) showed that with or without isoleucine, a *ridA* mutation decreased motility by ~35%. Finally, by increasing the production of 2AA, the motility was further decreased (Table 2, ln 11–16). In strains carrying a 2AA generating system (*ABcc1*), there are two copies of *ilvA*, the wild-type allele in the chromosome and the *ilvA219* allele under the control of an inducible *P*_*BAD*_ in the chromosome^34^. Isogenic *ridA* and *ridA*+ strains carrying the *ABcc1* construct were generated. In this experiment, without induction of *ilvA219*, the *ridA* mutant had 20% less motility than the wild-type strain. When expression of *ilvA219* was induced, the *ridA* mutant had a greater than 40% defect in relative motility. Addition of isoleucine partially restored motility, consistent with the presence of the wild-type IlvA, which is sensitive to allosteric inhibition. Taken together the data support the conclusion that like previously characterized phenotypes, the effect of the *ridA* mutation on motility is due to the accumulation of 2AA generated by IlvA.

**Table 2:** Accumulation of 2AA causes a motility defect of *ridA* mutant strains.

	Strain	Relevant genotype	Addition	Swim zone (mm)	% motility
1	DM9404	wild-type	None	19 ± 2	100
2	DM3480	*ridA*	None	11 ± 1	58
3	DM9404	wild-type	Thr	20 ± 1	100
4	DM3480	*ridA*	Thr	20 ± 1	100
5	DM9404	wild-type	Ile	21 ± 1	100
6	DM3480	*ridA*	Ile	23 ± 1	110
7	DM6947	*ilvA219*	Gly	19 ± 1	100
8	DM6946	*ridA ilvA219*	Gly	11 ± 1	58
9	DM6947	*ilvA219*	Gly, Ile	23 ± 1	100
10	DM6946	*ridA ilvA219*	Gly, Ile	16 ± 1	70
11	DM15035	*ABcc1*	Gly	21 ± 1	100
12	DM15036	*ridA ABcc1*	Gly	16 ± 1	76
13	DM15035	*ABcc1*	L-ara, Gly	25 ± 1	100
14	DM15036	*ridA ABcc1*	L-ara, Gly	14 ± 1	56
15	DM15035	*ABcc1*	L-ara, Ile, Gly	23 ± 2	100
16	DM15036	*ridA ABcc1*	L-ara, Ile, Gly	17 ± 1	74

The diameter of motility halos was determined on minimal glucose (lines 1–10) or glycerol (lines 11–16) motility medium (0.25% agar). When indicated, L-arabinose (0.2%), L-threonine (1 mM), L-isoleucine (1 mM), and/or L-glycine (0.67 mM) was added. Values shown as the average diameter and standard deviation (SD) of the growth zone of four biological replicates incubated for 20 h at 30 °C. The data for each biological replicate was the average of two technical replicates. Percent motility is the ratio of motility of the *ridA*-/*ridA*+ isogenic strains in each case.

### Lowered FlhD_4_C_2_ activity can explain reduced motility but not SPI expression

**Figure 5:**
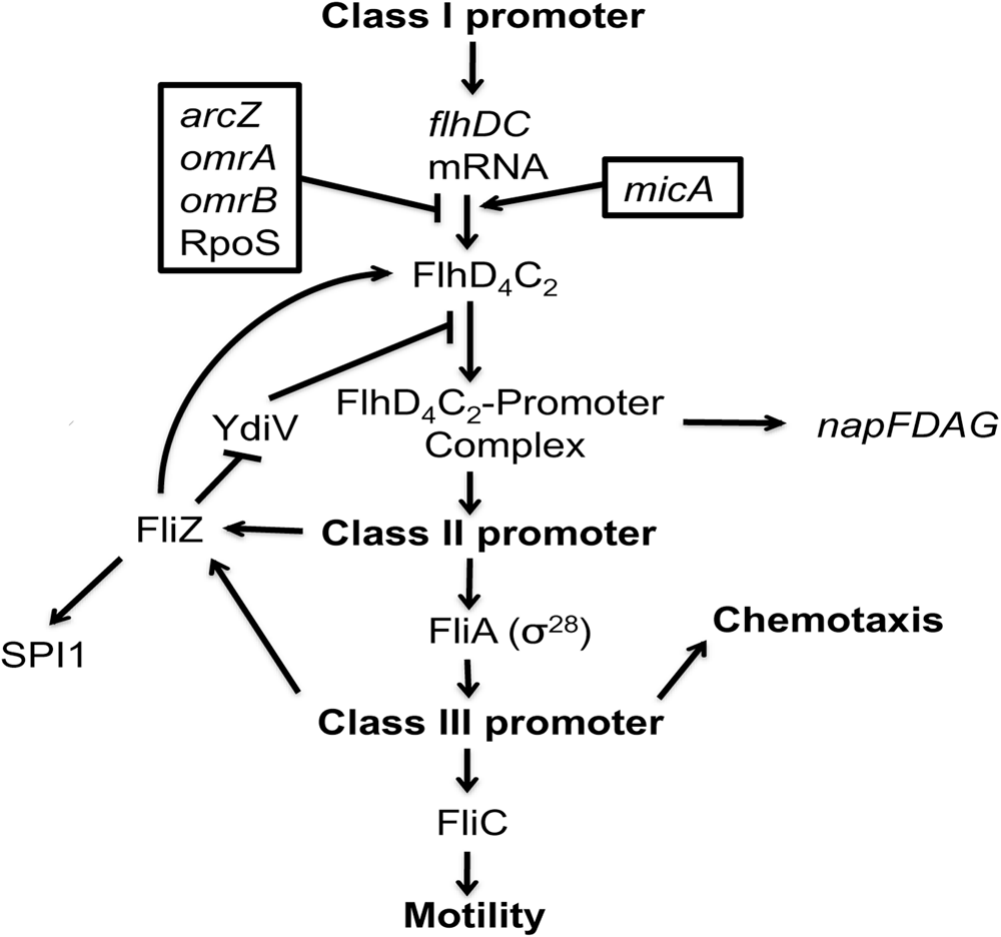
General regulatory cascade involving FlhD_4_C_2_. Schematic of the model for regulation involving FlhD_4_C_2_ in *E*. *coli* is depicted. Arrows and blocked arrows indicate positive and negative regulation, respectively. The four small Hfq-dependent non-coding regulatory RNAs *arcZ*, *omrA*, *omrB*, *and oxyS*, negatively regulate motility by binding with the 5′ untranslated region of *flhDC* and preventing translation^43^. YdiV acts as an anti-FlhD_4_C_2_ factor to disrupt promoter binding^37^. FliZ can counterbalance this affect by repressing *ydiV* expression as well as increasing FlhD_4_C_2_ protein abundance in an YdiV-independent manner^39^. FliZ also promotes increased Salmonella pathogenicity island 1 (SPI1) transcription^58^.

Biosynthesis of the *S*. *enterica* flagellar apparatus is controlled by a well-characterized regulatory cascade, and expression/activity of the master regulator FlhD_4_C_2_ is tightly regulated. Figure 5 schematically represents the relevant genes and their general position in this cascade. The *ridA* mutant had decreased expression of several operons that are directly, or indirectly, activated by the FlhD_4_C_2_ master regulator. These genes included *napFDAG*, *flg* and *fli* genes and those in SPI I, as indicated in Figure 2 ^35,36^. A simple scenario would suggest the function of the FlhD_4_C_2_ activator complex was reduced in a *ridA* mutant, resulting in the decreased expression of its regulon. However, the expression of *flhD* and *flhC* were significantly (FDR < 0.05) higher in the *ridA* strain (1.64, 1.58 –fold, respectively). These two results could be reconciled if the *ridA* mutation generated a post-transcriptional effect that resulted in differential function of FlhD_4_C_2_ in the two strains.

If accumulation of 2AA impacted one of the characterized regulators of *flhDC* translation or FlhD_4_C_2_-dependent promoter binding, elimination of the respective regulatory component would nullify the effect of the *ridA* mutation. Lesions in the small regulatory RNAs *omrBA*, *micA*, *arcZ*, *oxyS*, as well as *rpoS*, each of which post-transcriptionally regulate FlhD_4_C_2_, were introduced to isogenic *ridA* strains. The motility of the resulting strains was determined, and the data are in Table 3. Mutations in *arcZ* and *rpoS* caused no detectable defect in motility compared to wild-type and did not impact the effect of the *ridA* mutation (Table 3, ln 5–6, 13–14). Mutations in *micA*, *omrBA*, or *oxyS* decreased motility slightly (~20–25%) with a *ridA* mutation further decreasing the motility by ~30% (Table 3, ln 3–4, 7–10). The proteins YdiV and FliZ also regulate FlhD_4_C_2_ activity post-transcriptionally. YdiV acts on FlhD_4_C_2_ to disrupt promoter binding^37^ (Fig. 5). FliZ can repress *ydiV* expression, indirectly activating FlhD_4_C_2_ function^38^; as well as increase FlhD_4_C_2_ protein levels through an unknown YdiV-independent mechanism^39^ (Fig. 5). In the absence of FliZ, expression of genes activated by FlhD_4_C_2_ is reduced ~2-fold^31^, similar to the magnitude resulting in the *ridA* mutant (Fig. 2). Deletion of *fliZ* decreased motility of *S*. *enterica* by ~43%. Inclusion of a *ridA* mutation resulted in a strain that had 56% the motility of the parental *fliZ* strain (Table 3, ln 15–16) and suggested these two mutations had independent (additive) effects on motility. Deletion of *ydiV* slightly (~7%) increased motility of wild-type *S*. *enterica* and the impact of a *ridA* mutation introduced into this background was consistent with the ~30% decrease repeatedly observed (Table 3, ln 11–12). Taken together, these data did not support a role for 2AA acting via a known regulator of FlhD_4_C_2_ translation or promoter binding of the complex. Representative images for the motility measurements given in Tables 1–3 were compiled and incorporated into Supplementary Figure S1.

**Table 3:** Effect of *ridA* on motility is not via known regulators of FlhD_4_C_2_ activity.

	Strain	Relevant genotype	Swim zone (mm)	% motility	*ridA* effect (%)
1	DM9404	wild-type	15 ± 1	100	100
2	DM3480	*ridA*	11 ± 1	73	73
3	DM15509	*micA*	13 ± 1	86	100
4	DM15510	*micA ridA*	9 ± 1	60	69
5	DM15513	*arcZ*	15 ± 1	100	100
6	DM15514	*arcZ ridA*	9 ± 1	60	60
7	DM15507	*omrBA*	11 ± 1	73	100
8	DM15508	*omrBA ridA*	9 ± 1	60	82
9	DM15511	*oxyS*	12 ± 1	80	100
10	DM15512	*oxyS ridA*	9 ± 1	60	75
11	DM15319	*ydiV*	16 ± 1	107	100
12	DM15320	*ydiV ridA*	10 ± 1	67	63
13	DM15340	*rpoS*	15 ± 1	100	100
14	DM15341	*rpoS ridA*	10 ± 1	67	67
15	DM15505	*fliZ*	9 ± 1	60	100
16	DM15506	*fliZ ridA*	5 ± 1	33	56

Motility assays were performed as described in Materials and Methods, with the strains listed. Values shown are the average diameter and standard deviation (SD) of four biological replicates incubated for 20 h at 30 °C on minimal glucose (11 mM) L-glycine (0.67 mM) motility plates (0.25% agar). % motility is compared to the wild-type strain (line 1), ridA effect is the ratio of motility for the *ridA*+/*ridA*- isogenic strains.

**Figure 6:**
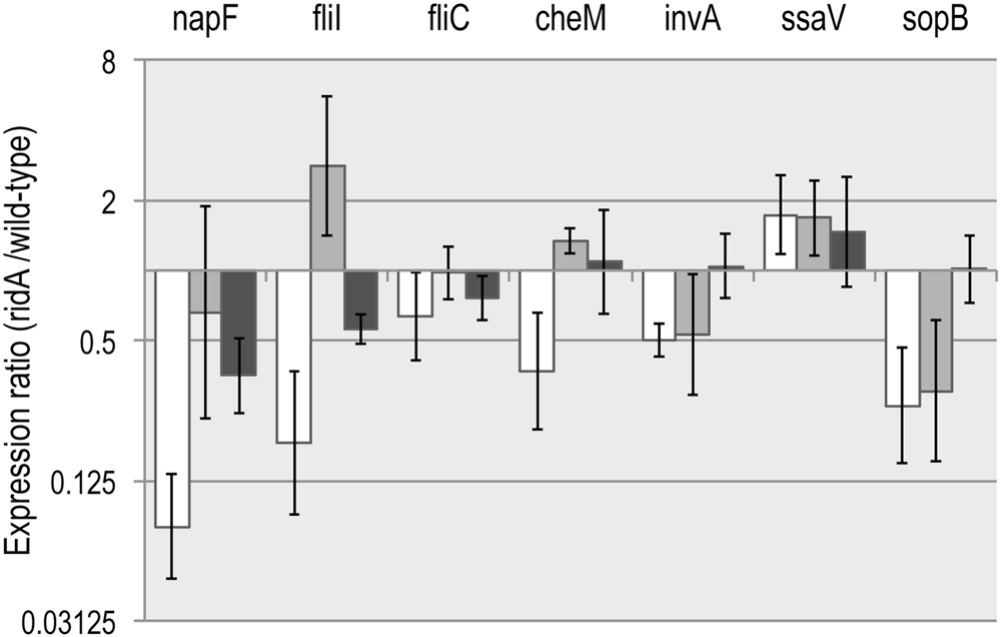
Differential expression is impacted by status of FlhD_4_C_2_ and growth medium. Expression of a subset of genes was monitored in a *ridA* strain (DM3480) relative to the isogenic wild-type strain (DM9404) by qRT-PCR. The strains were grown in minimal glucose medium (white bars) and minimal medium with 1 mM L-glycine (dark grey bars). Gene expression was similarly measured in an *flhDC ridA* strain (DM15817) relative to the isogenic *flhDC* strain (DM15818) when they were grown in minimal glucose medium (light grey bars). Error bars represent the 95% confidence interval.

To investigate the scope of *flhDC* involvement of the differential expression of motility, chemotaxis, and SPI-encoded genes in a *ridA* mutant, qRT-PCR compared the expression of representative loci (*napF*, *fliI*, *fliC*, *cheM*, *invA*, *ssaV*, and *sopB*) between a *ridA flhDC::cat* mutant (DM15818) and *flhDC::cat* wild-type (DM15817) (Fig. 6). Welch’s two-tailed t-test revealed that elimination of *flhDC* significantly (p-value < 0.05) changed the relative expression (*ridA*:wild-type) of the *napF*, *fliI*, and *cheM* genes. Strikingly, expression of *fliC* was not significantly (p < 0.05) different between the *flhDC* and *flhDC ridA* strains. These data supported a model that a single perturbation affecting FlhD_4_C_2_ activity, directly or indirectly, could be responsible for the expression pattern of genes involved in flagellar biosynthesis, nitrate reduction, and chemotaxis. It is formally possible that 2AA interacts directly with FlhD_4_C_2_ to reduce its activity, but since free 2AA is not known to damage enzymes lacking a PLP cofactor, a more likely model suggests a disruption of the metabolic network to which a post-transcriptional regulator of *flhDC* responds.

### Exogenous glycine overrides *ridA* mutant effect on SPI expression

The *flhDC* mutation did not alter the influence a *ridA* mutation had on transcription of genes located within SPI1 (*invA*), SPI2 (*ssaV*), and SPI5 (*sopB*). These data suggested that the accumulation of 2AA, caused by the *ridA* mutation, affected expression of genes in SPI1, SPI2, and SPI5 by an FlhD_4_C_2_-independent mechanism. Exogenous glycine eliminates many growth phenotypes of a *ridA* mutant because it bypasses the need for serine hydroxymethyl transferase (GlyA), which is a primary target of 2AA in *S*. *enterica* ^24^. qRT-PCR compared the expression of relevant loci (*napF*, *fliI*, *fliC*, *cheM*, *invA*, *ssaV*, and *sopB*) between *ridA* (DM3480) and wild-type (DM9404) strains grown on minimal medium supplemented with 1 mM glycine (Fig. 6, dark grey bars). Importantly, based on Welch’s two-tailed t-test, the relative expression (*ridA*:wild-type) of the *napF*, *fliI*, *cheM*, *invA*, and *sopB* loci was significantly different (p-value < 0.05) when the strains were grown in the presence vs. absence of glycine in the medium. Further, when considered as an isolated experiment, expression of *cheM*, *invA*, *ssaV*, and *sopB* was not significantly (p-value < 0.05) different between the *ridA* and wild-type backgrounds, when the cells were grown in medium with glycine. Taken together, these data suggested that exogenous glycine eliminated the effect of a *ridA* mutation on the expression of *cheM*, *invA*, *ssaV*, *and sopB*, but *napF*, *fliI*, and *fliC* expression was still significantly (p-value < 0.05) reduced in the *ridA* mutant. The latter result was consistent with the finding that glycine did not completely restore motility to the *ridA* mutant (Table 1, ln 3–4). The effect a *ridA* mutation had on the transcription of *napF*, *fliI*, and *cheM* was affected by both an *flhDC* deletion and glycine addition, suggesting both FlhD_4_C_2_ activity and glycine/5,10-methylene-tetrahydrofolate influence the expression of these genes^19,24^.

## Discussion

Differential gene expression was used to probe the global consequences of eliminating the RidA protein in *S*. *enterica*. The transcription dataset described herein made a few points. First, the biochemical defects previously identified in *ridA* mutants, with the possible exception of DadX, did not alter the transcription of genes in the respective metabolic pathways affected. This result was consistent with genetic data that *ridA* mutants have compromised enzyme activity but, in the absence of further perturbations, maintain a robust and functional metabolism. This observation is consistent with the capacity of microbes to adapt to environment changes by maintaining a robust metabolic network.

Second, the expression pattern of *ridA* vs. wild-type paralleled the expressional changes found with cells replicating in a macrophage, compared to the same strain grown *in vitro* ^7^. Similar expressional changes were seen for the genes associated with nitrate reduction, methionine biosynthesis, chemotaxis, flagellar biosynthesis, and genes encoded on SPI1, SPI2, and SPI5 (Fig. 2). These results hint at similarities between the metabolic state of *S*. *enterica* cells that accumulate 2AA and what the cells face during infection of a host. This similarity was emphasized by the coordinated response of genes encoded by the three different SPIs, which are all required for Salmonella enteropathogenicity^40,41^. Serine hydroxymethyl transferase (GlyA) is compromised, (i.e., glycine and C_1_ units are reduced) in strains lacking *ridA* ^24^. By adding glycine and restoring one-carbon unit abundance in a *ridA* mutant, the differential expression of these SPI loci was minimized. Further work will be needed to extend this correlation and determine whether the *ridA* paradigm can provide a model to extend the understanding of the intracellular environment and the factors influencing the response of *S*. *enterica* to the intracellular environment of the macrophage.

A third feature of the data was the reduced expression of flagellar biosynthesis genes, chemotaxis genes, and periplasmic nitrate reductase (*nap*) genes in a *ridA* mutant. Consistent with the expression pattern, *ridA* mutants had decreased motility, when compared to the parental strain. As with previously characterized phenotypes of a *ridA* mutant, the lowered motility was a consequence of 2AA accumulation, although the specific target and resulting mechanism were not clear. The only targets of 2AA characterized thus far are PLP-dependent enzymes, where the reactive 2AA enamine attacks the PLP in an active site to lock the respective enzyme in an inactive state^17,19,22^. There are no PLP-dependent enzymes directly involved in motility, suggesting either a new class of targets for 2AA, or an indirect response to a targeted PLP-dependent enzyme(s). The master activator, FlhD_4_C_2_, controls a significant number of the genes that are differentially regulated in a *ridA* mutant, including the periplasmic nitrate reductase (*nap*) associated genes. The qRT-PCR data showed that the transcriptional remodeling in a *ridA* mutant is multifaceted, even when only considering pathogenesis-associated genes (Fig. 5). Significantly, the differential expression of the *nap* operon, chemotaxis genes, and flagellar biosynthesis genes in a *ridA* mutant required an intact *flhDC* locus, while the differences in SPI gene expression were *flhDC*-independent. The *flhDC*-dependent link between the effect of 2AA on the *nap* operon and motility-associated gene expression suggested a need to coordinate nitrate reduction and motility^44^.

In total, the comparative transcription analyses herein identified a new motility phenotype that results from the accumulation of 2AA in *S*. *enterica* strains lacking *ridA*. The enamine deaminase activity of RidA is conserved across all domains of life, suggesting 2AA accumulation is a metabolic concern in other Enterobacteriaceae and an even wider set of organisms^16,23,34,45^ *E*. *coli* RidA has been reported to act as a chaperon to protect against reactive oxygen species (ROS) toxicity, specifically hypochlorite^42^. Significantly, ROS stress acts to repress the flagellar biosynthesis machinery^43^, making it a formal possibility that the chaperon role of RidA is involved in the motility defect described here. However, the genetic data linking the motility defect to 2AA production appeared to eliminate this possibility.

This study emphasized the ability of transcriptome analyses to provide global metabolic snapshots that may not result in a detectable growth phenotype. It is likely that some of the differentially expressed genes that failed to meet the arbitrary two-fold threshold will prove to be physiologically relevant. These data provided insights into the endogenous environment resulting from the accumulation of 2AA in *Salmonella* and uncovered areas of metabolism that may be targeted by metabolic stress caused by 2AA. Identifying additional enzymes that are affected by 2AA will require targeted genetic and biochemical approaches that are driven by global datasets, like the one described here.

## Methods

### Bacterial Strains, Plasmids, and Primers

Strains and plasmids used in this work were derivatives of *Salmonella enterica* subsp. *enterica* serovar Typhimurium LT2 and are listed in Table S2. Transductional crosses were carried out using the high-frequency general transducing mutant of bacteriophage P22 (HT105/1, *int*−201)^46^. New gene disruptions constructed during this study were made using the λ-Red recombinase system described by Datsenko and Wanner^47^ using the primers listed in Table S3, transduced into relevant genetic backgrounds by selecting chloramphenicol resistance, and verified by PCR. Plasmid pDM1439 was constructed using the high-efficiency cloning method^48^ to clone the LT2 *ridA* gene into plasmid pCV1. The resulting construct was confirmed by sequence. pCV1 is a modified pBAD24 plasmid containing BspQ1 sites, conferring ampicillin resistance, and the cloned gene’s expression is inducible by L-(+)-arabinose^49^. Gene-specific primers for cloning and qRT-PCR analysis were synthesized by Integrated DNA Technologies, Inc. (Coralville, IA) and are listed in Table S3. Primers were designed using Primer 3 software, and evaluated for specificity by melting curve analysis using *S*. *enterica* LT2 gDNA prior to running the qPCR experiment.

### Culture Media and Chemicals

Minimal medium was no-carbon E medium (NCE) supplemented with 1 mM MgSO_4_^50^, trace minerals^51^, and 11 mM D-glucose as the sole carbon source. L-(+)-arabinose was added to a final concentration of 0.2 g/L when needed. Difco nutrient browth (NB) (8 g/L) containing NaCL (5 g/L) was used as rich medium. Difco BiTek agar (15 g/L) was added for solid medium. Ampicillin was added when required for plasmid maintenance to rich and minimal medium at 150 and 7.5 μg/mL, respectively. Chloramphenicol (20 μg/mL) and Ampicillin (150 μg/mL) were used in rich medium, during strain construction. All chemicals were obtained from Sigma-Aldrich Chemical Company (St. Louis, MO).

### RNA isolation

Relevant strains were grown overnight with shaking at 37°C in quadruplicate (RNA-seq) or triplicate (qRT-PCR) in 2 mL NB. Following incubation, strains we diluted 1:100 into 5 mL fresh minimal medium, containing any additional supplements indicated in the text, and allowed to grow with shaking at 37°C to an optical density at 650 nm (OD_650_) of 0.6. Each 5 mL sample was quickly centrifuged at 16,000 × *g* in 1.5 mL Eppendorf tubes, supernatant removed and pellets flash-frozen in liquid nitrogen and kept on dry ice. Total RNA was extracted using the RNA*snap* ^TM^ method^52^. Pellets were resuspended in 110 μl boil solution (95% [vol/vol] RNA-grade formamide, 18 mM EDTA, 0.025% [wt/vol] SDS, 1% 2-mercaptoethanol in UltraPure^TM^ (ThermoFisher) distilled water). Samples were incubated at 95°C for 7 min before centrifuging at 16,000 × *g* for 5 min while still hot and 100 μl supernatant was transferred to a new 1.5 mL Eppendorf tube. 300 μl UltraPure^TM^ water and 40 μl sodium acetate (3 M [pH5.2]) was added and mixed into each sample before adding 400 μl ice-cold ethanol (100%) and incubating on ice 15 min. Samples were centrifuged at 16,000 × *g* for 15 min at 4°C and ethanol was decanted. 400 μl ethanol (70% [vol/vol]) was added and pellets were immediately centrifuged at 12,000 × *g* for 10 min at 4°C before decanting ethanol and pellets were allowed to dry 20 min at 25°C. DNA/RNA pellets were resuspended in 90 μl UltraPure^TM^ water at 4°C overnight prior to treatment with RNase-free Turbo DNase (Ambion), and being precipitated once more by sodium acetate-ethanol treatment. Resulting RNA was resuspended at 4°C for 3 h and stored at −80°C until use.

### RNA-Seq expression profiling and data analysis

The total RNA was extracted from four independent replicates of *S*. *enterica* wild-type and *ridA* mutant strains. In each of the resulting eight samples, rRNA was removed from 5 μg total RNA using the Ribo-Zero rRNA removal kit (Bacteria; Illumina) following manufacturer’s recommendations. A cDNA library was created from samples containing < 10% rRNA contamination, as measured by the RNA 6000 pico kit for the Agilent 2100 bioanalyzer, and sequenced by the University of Georgia Genomics Facility (GGF) from these RNA samples. Specifically, one hundred nanograms of the rRNA-free RNA was used to create each sequencing library using the KAPA stranded RNA-seq kit (KAPA Biosystems). The RNA libraries were sequenced on an Illumina NextSeq. 500 (150 cycles) Mid Output Flowcell in the paired-end mode with a read length of 75 bp. Processing and differential expression determination of the sequencing data were performed by the Georgia Advanced Computing Resource Center (GACRC) at the University of Georgia. Adaptor removal and quality trimming (Phred score, ~20) of the raw reads were done using FastQC (http://www.bioinformatics.bbsrc.ac.uk/projects/fastqc/) and Trimmomatic (Usadel). The trimmed reads were mapped to *Salmonella enterica* subsp. Enterica serovar Typhimurium str. LT2 chromosome, complete genome (RefSeq Accession Number: NC_003197.1) and read counts were generated using the prokaryotic-tailored gene alignment tool EDGE-Pro^53^. Genes differentially expressed (false discovery rate, FDR < 0.05) between the respective wild-type and *ridA* strains of *S*. *enterica* were identified using DEseq^27,30,54^. Briefly, an FDR < 0.05 cutoff controls the expected proportion of falsely discovered differentially expressed genes (false positives) to 5% (21 genes) of the identified positive hits meeting this threshold (413 genes). The procedures used by DEseq software to determine the FDR value for each gene is described by Benjamini and Yekutieli^27^.

### qRT-PCR

The total RNA from three independent replicates of each *S*. *enterica* strain tested, as described in the text, was extracted. An aliquot of each sample was sent to GGF for quality control analysis and quantification using the RNA 6000 nano kit for the Agilent 2100 bioanalyzer and only samples with an RNA integrity number (RIN) over 5.0 were used^55^. DNase-treated RNA (600ng) was subjected to first strand cDNA synthesis, using the iScript cDNA synthesis kit (Bio-Rad Laboratories) according to the manufacturer’s protocol. PCRs were performed in an Applied Biosystems 7500 Fast real-time (RT) PCR system. Each reaction was carried out in a total volume of 20 μl on a 96-well optical reaction plate (Applied Biosystems) containing 10 μl FastStart Universal SYBR green Master (ROX) mix (Roche Applied Science), 8 ng cDNA, and two gene-specific primers at a final concentration of 0.5 μM each. The real-time cycling conditions were as follows: 95°C for 20 s, and 40 cycles of 95°C for 3 s and 60°C for 30 s. Melting-curve analysis verified that each reaction mixture contained a single PCR product. The threshold cycle (CT) values of *gyrB* and *rpoB* were used as internal controls^56,57^. Fold changes were calculated using the comparative threshold cycle (ΔΔCT) method^29^. Briefly, the fold-change in gene expression was calculated using the formula: *ridA*/wild-type = 2^ΔΔCT^, where ΔΔCT = ΔCT_*ridA*_ - ΔCT_wild-type_ and ΔCT = CT_target gene_ - CT_normalization gene (*gyrB*)_. Gaussian error propagation was used to determine the standard error of the mean (SEM) for ΔΔCT from the ΔCT SEM values, and this value used to calculate the 95% confidence interval for each gene. Welch’s two-tailed t-test was performed on log_2_ transformed 2^ΔΔCT^ values, using GraphPad Prism 6.0b software. To ensure *gyrB* was constant under the conditions tested, the relative fold change for the internal control *rpoB*, whose expression was expected to remain constant across treatments, was calculated as described above. Only those samples with an *rpoB* fold change between 0.8 and 1.25 were used.

### Motility Assays

Motility was monitored on minimal medium motility plates (0.25% agar) using either D-glucose (11 mM) or glycerol (20 mM) as the sole carbon source. The plates were prepared in the morning and allowed to solidify at room temperature un-stacked on the benchtop ~6 h before use in the evening. *S*. *enterica* cells were grown to full-density in LB and 1 μl of the culture was inoculated onto the plate and incubated at 30 °C inside of a sealed 6 quart plastic Sterilite container to maintain constant moisture content. Motility halos were measured after 20 h using a ruler with markings every mm; if the halo diameter fell between mm markings, the recorded halo diameter was rounded to the nearest marking. Technical replicates (i.e., plates) were prepared from each of four biological replicates and the data were averaged and expressed as mean (mm) ± SD.

### Data Availability

Sequencing data were deposited at the NCBI Gene Expression Omnibus (GSE103146), and can be found at https://www.ncbi.nlm.nih.gov/geo/query/acc.cgi?acc=GSE103146. All other datasets generated in the current study are available from the corresponding author on reasonable request.

## Electronic supplementary material

Supplementary information

Supplementary Table 1
