# Supplementary material for: Endogenously generated 2-aminoacrylate inhibits motility in Salmonella enterica
Source: Sci Rep. 2017 Oct 11;7:12971. doi: 10.1038/s41598-017-13030-x (PMC5636819; doi:10.1038/s41598-017-13030-x)
Supplement: Supplementary file 1 — Supplementary information [file 41598_2017_13030_MOESM1_ESM.pdf]

Endogenously generated 2-aminoacrylate inhibits motility in *Salmonella enterica*

Andrew J. Borchert[1] and Diana M. Downs[1*]

[1]Department of Microbiology, University of Georgia, Athens, GA, USA

Supplementary Materials

Table S2. Bacterial strains and plasmids.		
Strain	Genotype[a]	Source
DM9404	Wild-type (isogenic to DM3480)	Lab collection
DM3480	*ridA3*::MudJ[b]	Lab collection
DM6946	*ilvA219 ridA3::*MudJ	Lab collection
DM6947	*ilvA219*	Lab collection
DM15035	*ABcc1*[c]	Lab collection
DM15036	*ridA*3::MudJ *ABcc1*	Lab collection
DM15418	WT / pCV1	This study
DM15419	*ridA*3::MudJ / pCV1	This study
DM15420	WT / pDM1439	This study
DM15421	*ridA3*::MudJ/ pDM1439	This study
DM15319	*ydiV*::*cat*[d]	This study
DM15320	*ridA3::*MudJ *ydiV::cat*	This study
DM15340	*rpoS*1117::*amp*	This study
DM15341	*ridA3::*MudJ *rpoS*1117*::amp*	This study
DM15505	*fliZ*::*cat*	This study
DM15506	*ridA3::*MudJ *fliZ::cat*	This study
DM15507	*omrBA*::*cat*	This study
DM15508	*ridA3::*MudJ *omrBA*::*cat*	This study
DM15509	*micA*::*cat*	This study
DM15510	*ridA3::*MudJ *micA*::*cat*	This study
DM15511	*oxyS*::*cat*	This study
DM15512	*ridA3::*MudJ *oxyS*::*cat*	This study
DM15513	*arcZ*::*cat*	This study
DM15514	*ridA3::*MudJ *arcZ*::*cat*	This study

DM15817	*flhDC*::*cat*	This study
DM15818	*ridA3*::MudJ *flhDC*::*cat*	This study
**Plasmid**	**Description**	**Source**
pCV1	*BspQ*I modified pBAD24	Lab collection
pDM1439	pCV1-*ridA*[e]	Lab collection

[a]All strains were derived from *Salmaonella enterica* serovar Typhimurium LT2

[b]MudJ refers to the MudJ1734 transposon [Castilho, Olfson, and Casadaban,1984].

[c]*ABcc1* is a chromosomal cassette (*araBAD*::P_{BAD}-*ilvA219 cat*) that has the *ilvA219* allele under the inducible control of the P_{BAD} promoter and encodes chloramphenicol resistance [Borchert, 2017].

[d]*cat* alleles are insertion deletions of the relevant loci as described in the Materials and Methods.

[e]Plasmid constructed using pCV1 [Vandrisse, 2016].

**Table S3. Primers used in this study.**	
**Primer Name**	**Primer Sequence 5' → 3'**
**Strain construction**	
LT2 Wanner *ydiV* 5'	ACTGGATGGCGAATAGCGCCCTAACCATGGGACTGG CGTAGTGTAGGCTGGAGCTGCTTC
LT2 Wanner *ydiV* 3'	GCAATTCATCGCGCCAACGGGCAGTAAAAGACAGGG TCATCATATGAATATCCTCCTTAG
LT2 Wanner *fliZ* 5'	CGAAAAGTGCCGCACAACGTATAGACTACCAGGAGT TCTCGTGTAGGCTGGAGCTGCTTC
LT2 Wanner *fliZ* 3'	CACGTTTCACCAACACGACTCTGCTACATCTTATGCTT TTCATATGAATATCCTCCTTAG
LT2 Wanner *omrBA* 5'	GTGACCTGCACGGGAGAAATGCGTCTGTAAAGTGAC AATAGTGTAGGCTGGAGCTGCTTC
LT2 Wanner *omrBA* 3'	TAACAGGAGCGATAGCAAAATAGGTTCGAAAAAAAA CCTGCATATGAATATCCTCCTTAG
LT2 Wanner *micA* 5'	ATAAACTGAACTCTTTGTTCCGGGGCGAGTCTGAGTA TATGTGTAGGCTGGAGCTGCTTC
LT2 Wanner *micA* 3'	CGAGCCGTTTGCCGCGTGGCTTGCAAAACACGCCTGA CCCCATATGAATATCCTCCTTAG
LT2 Wanner *oxyS* 5'	CGATTATCCCTATCAACCTTTCTGATTAATAATACATC ACGTGTAGGCTGGAGCTGCTTC
LT2 Wanner *oxyS* 3'	CCGAGCATCAGCGACGCCGGGTTTTTTACGCAAAAA AAACATATGAATATCCTCCTTAG
LT2 Wanner *arcZ* 5'	TTCATGTAACAAATCATTTAGGATTTGCTATCTTAACT GCGTGTAGGCTGGAGCTGCTTC
LT2 Wanner *arcZ* 3'	GGCAAACGCGGAAAAAAAATGACCCCGGTTTGACCG GGGTCATATGAATATCCTCCTTAG
LT2 Wanner *flhDC* 5'	GTGCGGCTACGTCGCACAAAAATAAAGTTGGTTATTC TGGGTGTAGGCTGGAGCTGCTTC
LT2 Wanner *flhDC* 3'	TCTGTTCATCCAGCAGTTGTGGAATAATATCGGCAGC ATCCATATGAATATCCTCCTTAG
**Cloning**	
LT2 *ridA* cloned into pCV1 5'	NNGCTCTTCNTTCATGAGCAAAACTATCGC
LT2 *ridA* cloned into pCV1 3'	NNGCTCTTCNTTATTAGCGACGAACAGC
**Sequence verification primers**	
LT2 check *ydiV* 5'	GGAACCGGTTCACCGGTAAA
LT2 check *ydiV* 3'	AACGGTGTGGGTAAAAGCGC
LT2 check *fliZ* 5'	TTGGCGCGGTACTGGAA
LT2 check *fliZ* 3'	TCATAGTAACCCCGAATATTGCC
LT2 check *omrBA* 5'	CGCTTACATTACGCCAGT
LT2 check *omrBA* 3'	TTCAACTCCCTTTGGCC
LT2 check *micA* 5'	TTAGTCACCTCCGATAAT
LT2 check *micA* 3'	GTTTATTGCTGAACGTGA
LT2 check *oxyS* 5'	TATTCATCCTCCGTCGC
LT2 check *oxyS* 3'	GCCTTATAAGCATAGCGC

LT2 check *arcZ* 5’	GGTGAACATCCTCCTGC
LT2 check *arcZ* 3’	CAGCAAATCCAGTCGC
LT2 check *flhDC* 5’	CTAAAGGTAAAATAAAAGCG
LT2 check *flhDC* 3’	CATCCTTCCGCTGTTGAC
pBAD sequencing 5’	TACCTGACGCTTTTTATCGC
pBAD sequencing 3’	GAAAATCTTCTCTCATCCGC
**RT-qPCR primers**	
LT2 *dadX* qRT-PCR 5’	CGGGTGAAAGGGTAGGCTAT
LT2 *dadX* qRT-PCR 3’	GTGGATAGCCATCGGCATAA
LT2 *fliI* qRT-PCR 5’	GACCAACAAGATCCCATTGC
LT2 *fliI* qRT-PCR 3’	CGATATCAATGGCCGGATAG
LT2 *metH* qRT-PCR 5’	ACATTGCCCGTGTACCAATC
LT2 *metH* qRT-PCR 3’	GACGATGCCTTTACCCTGAA
LT2 *napF* qRT-PCR 5’	GCCAGGACAGTTGTGAACCT
LT2 *napF* qRT-PCR 3’	TACAGGCCTGCGAGTCAAG
LT2 *thiF* qRT-PCR 5’	GCCAGCTAATGGTCCTCACA
LT2 *thiF* qRT-PCR 3’	GTACGGCAGTTGCGTTCAG
LT2 *sdaC* qRT-PCR 5’	AAGGCTTTAACGGGATGGTG
LT2 *sdaC* qRT-PCR 3’	GTGGTGACCAGCATGAACAG
LT2 *fliC* qRT-PCR 5’	GCAAGTAAAGCCGAAGGTCA
LT2 *fliC* qRT-PCR 3’	GTCAACCTGTGCCAAAGCAG
LT2 *cheM* qRT-PCR 5’	GTAACGCAGGTTCGTGAAGG
LT2 *cheM* qRT-PCR 3’	TGTTCGGTACGGGAAGAGAG
LT2 *invA* qRT-PCR 5’	GTCCTCCGCCCTGTCTACTT
LT2 *invA* qRT-PCR 3’	CGGCACTAATCGCAATCAAC
LT2 *ssaV* qRT-PCR 5’	TCGGCACCTTAATTGACTGG
LT2 *ssaV* qRT-PCR 3’	CGGATTAAGACGACGCAGAA
LT2 *sopB* qRT-PCR 5’	CGGGCATCACTATACCAACAC
LT2 *sopB* qRT-PCR 3’	CCCTCATAAGCACTGGGAAA

Supplementary Figure S1: Representative motility assay photos for data presented in Tables 1-3.

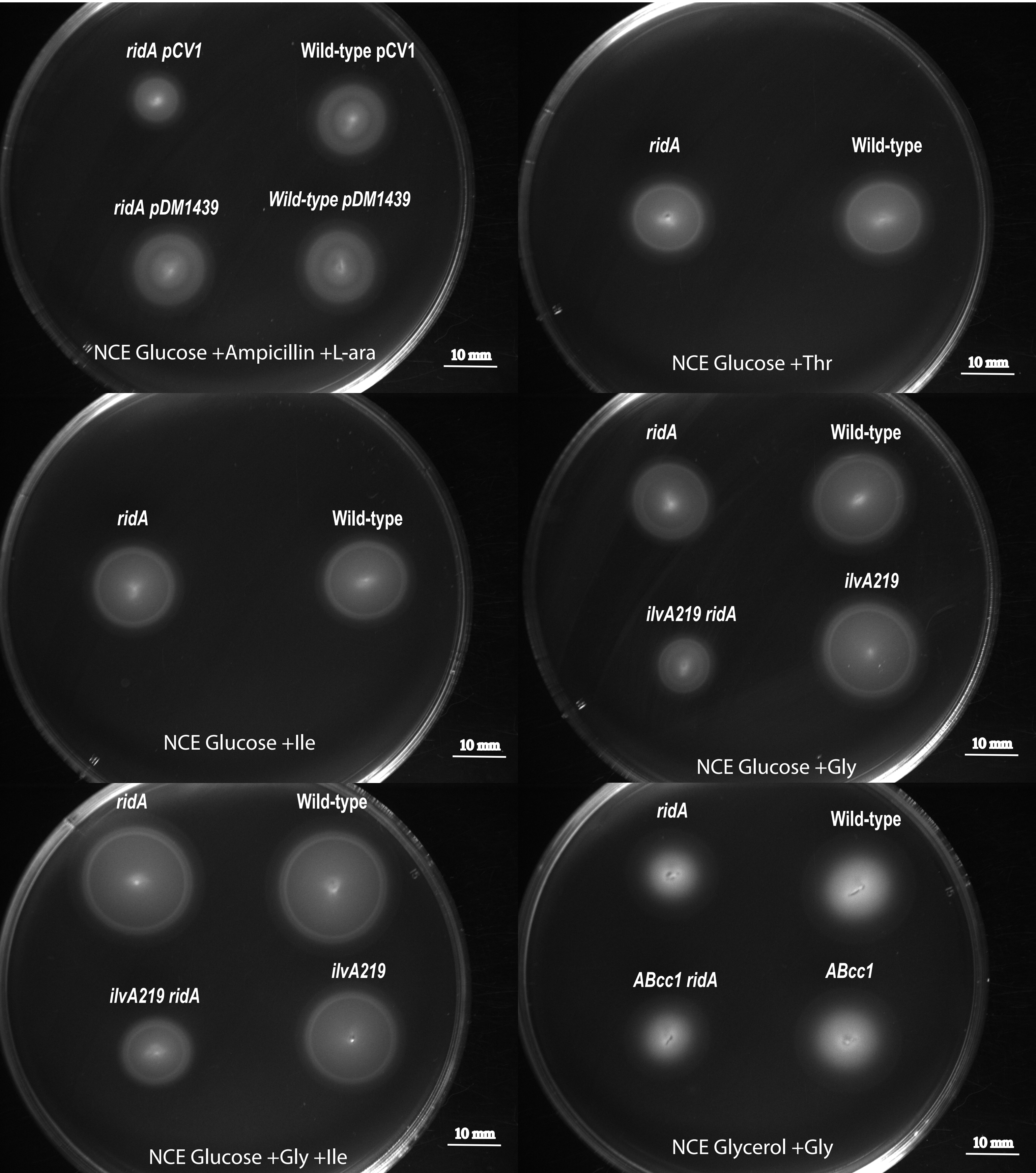

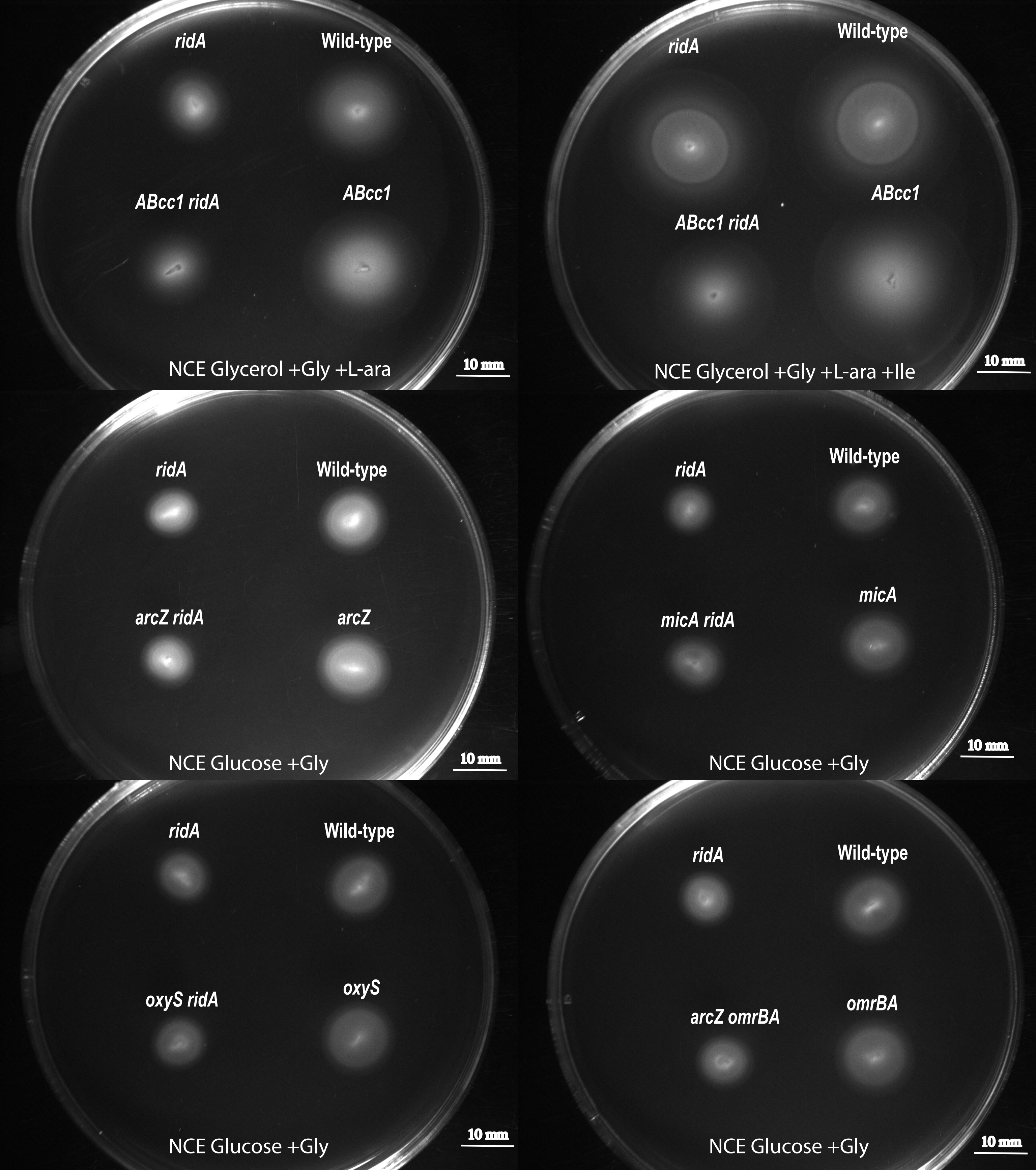

ridA
Wild-type
ABcc1 ridA
ABcc1
NCE Glycerol +Gly +L-ara
10 mm
ridA
Wild-type
ABcc1 ridA
ABcc1
NCE Glycerol +Gly +L-ara +Ile
10 mm
ridA
Wild-type
arcZ ridA
arcZ
NCE Glucose +Gly
10 mm
ridA
Wild-type
micA ridA
micA
NCE Glucose +Gly
10 mm
ridA
Wild-type
oxyS ridA
oxyS
NCE Glucose +Gly
10 mm
ridA
Wild-type
arcZ omrBA
omrBA
NCE Glucose +Gly
10 mm

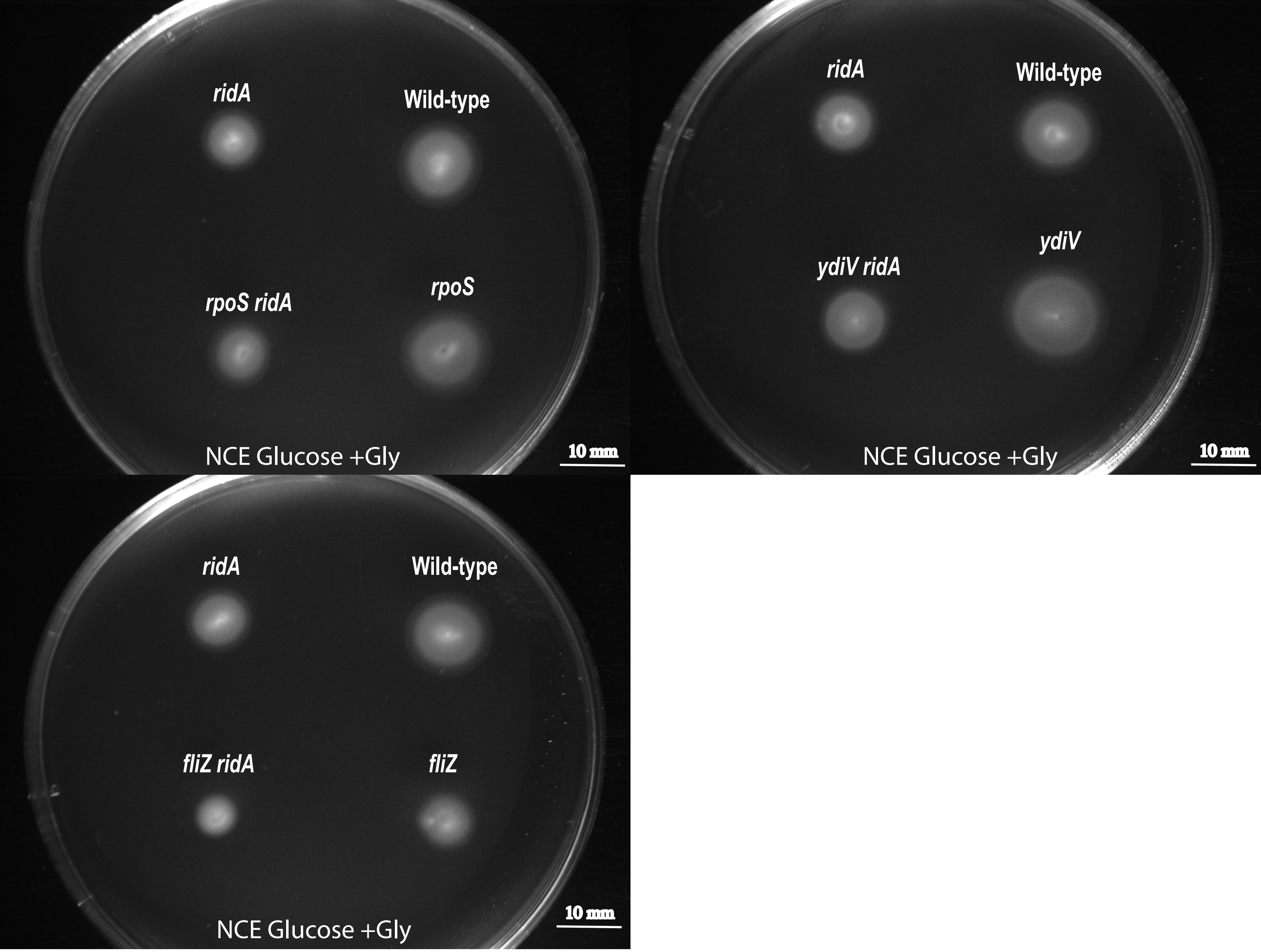
ridA
Wild-type
rpoS ridA
rpoS
NCE Glucose +Gly
10 mm
ridA
Wild-type
ydiV ridA
ydiV
NCE Glucose +Gly
10 mm
ridA
Wild-type
fliZ ridA
fliZ
NCE Glucose +Gly
10 mm
